# Rational design matrix materials for organoid development and application in biomedicine

**DOI:** 10.1093/rb/rbaf038

**Published:** 2025-05-14

**Authors:** Yue Huang, Xiaoyu Zhang, Wanjun Zhang, Jinglong Tang, Jing Liu

**Affiliations:** CAS Key Laboratory for Biomedical Effects of Nanomaterials and Nanosafety & CAS Center for Excellence in Nanoscience, National Center for Nanoscience and Technology of China, University of Chinese Academy of Sciences, Beijing 100190, China; CAS Key Laboratory for Biomedical Effects of Nanomaterials and Nanosafety & CAS Center for Excellence in Nanoscience, National Center for Nanoscience and Technology of China, University of Chinese Academy of Sciences, Beijing 100190, China; Department of Occupational and Environmental Health, School of Public Health, Qingdao University, Qingdao 266021, China; Department of Occupational and Environmental Health, School of Public Health, Qingdao University, Qingdao 266021, China; CAS Key Laboratory for Biomedical Effects of Nanomaterials and Nanosafety & CAS Center for Excellence in Nanoscience, National Center for Nanoscience and Technology of China, University of Chinese Academy of Sciences, Beijing 100190, China

**Keywords:** organoid, hydrogels, biomedicine, cancer, drug-screening

## Abstract

Organoids are three-dimensional tissue analogues grown *in vitro*. Although they are not human organs in the strict sense, they can mimic the structure and function of tissues *in vivo* to the maximum extent, and have broad application prospects in the fields of organ development, personalized medicine, regenerative medicine, disease modeling, drug screening, gene editing, etc. There is even hope that organoids can replace experimental animals for preclinical testing, which will greatly shorten the cycle of preclinical testing and improve its efficiency. Nowadays, Matrigel remains the predominant substitute for organoid culture systems. At the same time, new extracellular matrix or inspired polymer materials with tunable and optimized biochemical and biophysical properties continue to emerge, which are of great significance for efficient and high-level cultivation of organoids. In this review, we critically evaluate how mechanobiological signaling dynamics at the cell–matrix interface inform the rational engineering of biomimetic extracellular matrices to achieve standardized and phenotypically regulated patient-derived organoid cultures. Then, we systematically classify hydrogel-based matrices encompassing natural, biohybrid, synthetic, protein-engineered and DNA crosslinked matrix systems by their biocompatibility and functional compatibility. Focusing on cancer oncogenesis and progression research, drug development and personalized medicine, we highlight biomimetic hydrogel innovations that recapitulate tumor organoids development. By summarizing the obstacles that hinder the development of organoid hydrogels, we hope to provide an outlook on the future directions for the development of organoid hydrogels and promote the application of organoids in the field of biomedicine.

## Graphical Abstract

**Figure:**
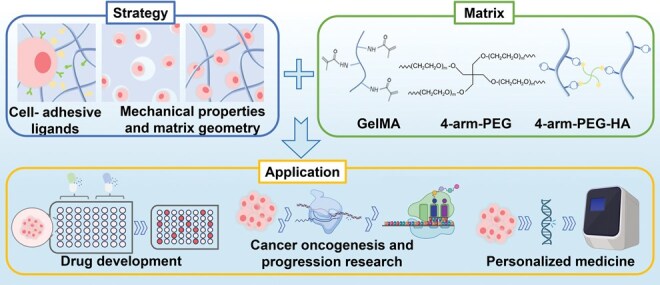

## Introduction 

Organoids are three-dimensional cell aggregates of pluripotent stem cells or tissue-derived progenitor cells that can be spontaneously assembled into morphologies of the corresponding organ tissue. Cells are artificially induced to adhere and differentiate into complex spatial structures that exhibit physiological responses and functional properties similar to those of the target tissue [1]. Organoids can not only phenotypically and functionally mimic the tissue but also have a relatively stable genetic profile, allowing for prolonged culture *in vitro*. In contrast to traditional two-dimensional culture models, organoids represent an innovative technology capable of recapitulating the physiological processes of an entire organism, with the advantages of more closely resembling physiological cell composition and behavior, more stable genomes and suitability for biological transfection and high-throughput screening. In contrast to animal models, organoid models are easier to perform and can be used to study disease initiation and progression [2]. As such, they hold promise for a wide range of applications in organ development, personalized medicine, regenerative medicine, disease modeling, drug screening and gene editing.

Organoids can be mainly divided into two main types from the perspective of stem cell origin: (1) pluripotent embryonic stem cells (ESCs) or their synthetic induced pluripotent stem cells (iPSCs)—derived organoids and (2) organ-restricted adult stem cells (ASCs)—derived organoids. Although ESCs and iPSCs have stronger differentiation ability than ASCs, there is no essential difference in the organoids formed. Also rapidly evolving in synchrony with organoids are *in vitro* cell culture techniques. Two-dimensional cell culture models are simple and have a high throughput but they fail to capture the physiological complexity of entire tissues and organisms, many beneficial properties of stem cells might be lowered or even lost, whereupon 3D cell culture systems are gradually established [3]. 3D cell culture refers to providing more complete cell-to-cell and cell-to-matrix interactions to better mimic the natural environment in which stem cells reside [4]. Although 3D cell culture manipulation is more complex and the conditions are more demanding, the use of 3D culture techniques has become more common in basic and translational research [5–7].

The extracellular matrix (ECM) has many functions and is a major component of the cellular microenvironment, participating in the most basic cellular behaviors, from cell proliferation, adhesion and migration to cell differentiation and cell death [8–10]. Hydrogels are biomaterials that are used in cell culture systems to imitate critical features of a natural extracellular matrix. Hence, hydrogels, derived from or designed with inspiration from ECMs, the biopolymeric structures that surround cells in tissues, are critical enablers in 3D culture of organoids [11, 12]. Matrigel, currently commercially available, is a basement-membrane matrix extracted from Engelbreth–Holm–Swarm mouse sarcomas and used for cell culture for over 40 years [13]. But intrinsic batch-to-batch heterogeneity in Matrigelmanifested as non-uniform biophysical stiffness gradients and stochastic ligand densities—compromises experimental reproducibility, impeding precise spatiotemporal control over mechanotransductive signaling required to elicit predictable morphogenetic or oncogenic phenotypes in engineered organoid systems [14]. Therefore, using chemical strategies to synthesize hydrogels with well-defined physical properties and biological functions in a controllable manner is critical for organoid culture [11].

The interaction between cells and matrix materials is the key factor affecting cell culture. From this point on, we briefly introduce the influence of mechanical properties and structure of materials on cells. After that, the most advanced engineering materials for organoid culture were discussed, aiming to find the commonness of these materials in performance and preparation methods, and provide ideas for the development of next-generation materials. Based on the application of organoids in cancer modeling, personalized medicine and drug development, we propose several challenges in the further development of organoids, which may be helpful for the future development.

## Cell–matrix interactions

**Figure 1.**
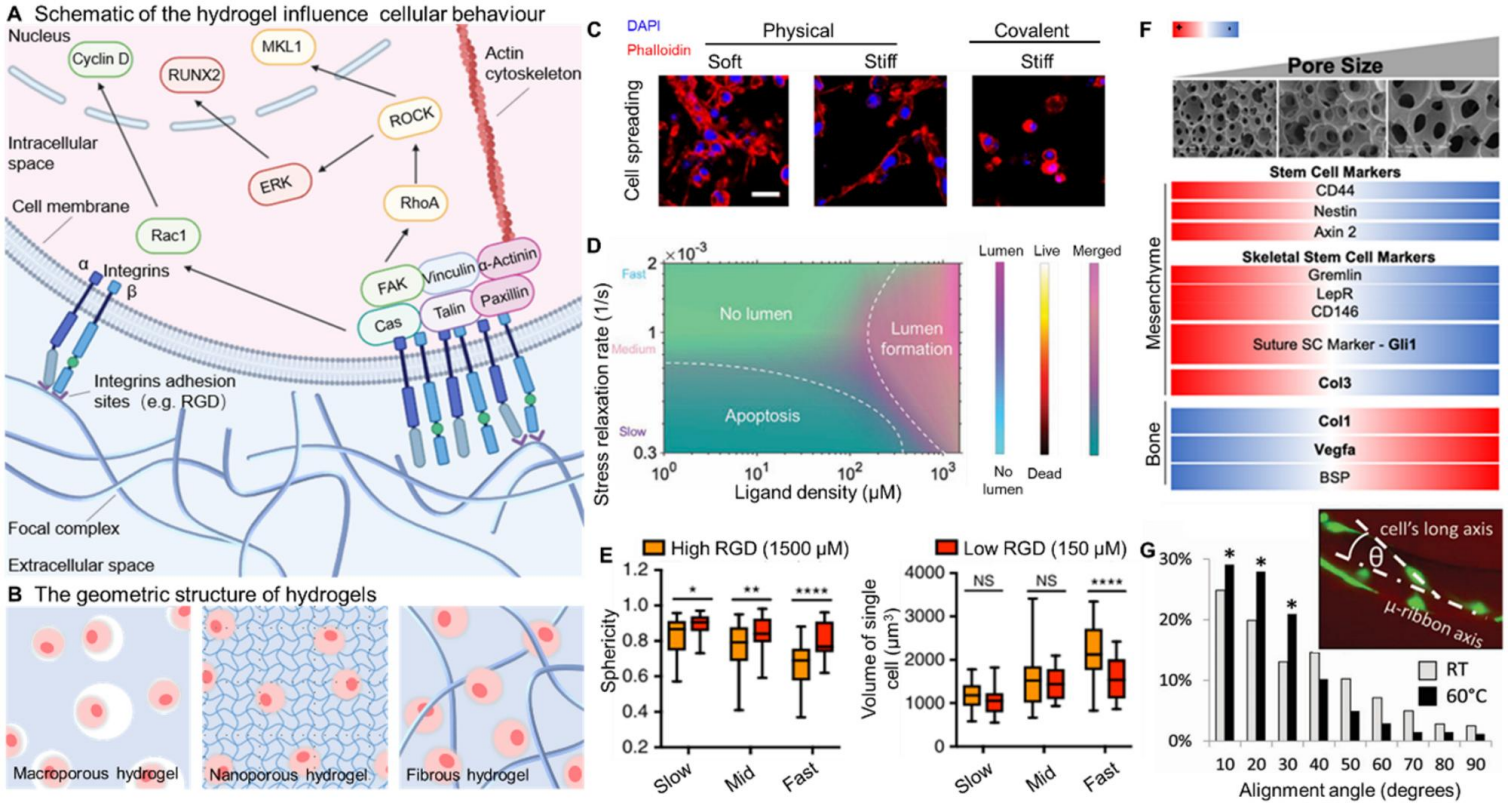
Cell–matrix interactions guiding the hydrogel design. (**A**) Schematic of the pathway of hydrogel mechanical characteristics affecting cell behavior. (**B**) The geometric structure of hydrogel influences the perception of external mechanics of cells. (**C**) Physically crosslinked alginate hydrogels facilitate matrix remodelling (cell spreading) [15]. (**D**) Summary of effect of matrix stress relaxation and ligand density on hiPSC behavior [16]. (**E**) Quantification of cell volume and sphericity of MSCs cultured in hydrogels with different rate of stress relaxation for 7 days with a RGD density of 150 µM and 1500 µM [17]. (**F**) Pearson similarity matrices were calculated for each heatmap to visualize and deduce trends in the data by correlating pore size, cell type and gene [18]. (**G**) Histogram data shows enhanced cell alignment on microribbons dried at 60°C than RT [19].

Ascertaining the interactions between cells and matrix is critical for designing and preparing engineered materials for organoids. The interaction between cells and the culture substrate is very complex. Living cells can sense and respond to a wide range of external signals, both chemical and physical, and they can integrate and analyze this information (Figure 1A). As a result, they can change their morphologies, dynamics, behaviors and ultimately fate [20]. Studying cell–substrate interactions and mimicking natural extracellular matrix properties are effective methods to prepare substrates that can be used for organoid culture *in vitro*. Now, available 2D cell cultures are based on tissue culture plastic dishes with a stiffness in the gigapascal range, which are considerably different from the native extracellular environment. Real ECM is a viscoelastic three-dimensional scaffold that provides structural and biochemical support for cells [21]. It contains a variety of proteins and polysaccharides, such as collagen I and III, hyaluronic acid (HA) and proteoglycan (PG), which can interact with cells. These chemical and physical properties of ECM control the cell spreading, proliferation and differentiation [22]. This is also the reason why 2D cell culture mostly stays at the flat and stretched monolayer level and cannot extend to the organoid level. Three-dimensional culture can simulate the ECM by regulating the chemical and mechanical properties and geometric configuration of the material (Figure 1B), better establish the cell–matrix interactions and promote the proliferation and differentiation of cells *in vitro* [23, 24].

### Cell-adhesive ligands

Native ECM is mainly composed of collagen, noncollagenous protein (e.g. laminin, fibronectin, etc.), elastin, PG and aminoglycans [25]. Among them, collagen, laminin and fibronectin are the ligands of cell-ECM. This interaction is mainly mediated by integrins, the main cell surface receptors [20, 26]. In addition to integrins, there are many other adhesion receptors on the cell surface, corresponding to different adhesion ligands in the ECM. The cell–matrix adhesion complexes (CMAC) can control the flow of the information (including transmission directions and details) [25] between the cell and the ECM, which in turn controls the fate of the cell, such as cell migration [26], cell proliferation [27], and differentiation [8]. Therefore, it is of great significance to construct matrix materials with cell adhesion ligands for 3D cell culture.

Based on the strategy of promoting the formation of CMAC between matrix and cell, many peptides have been used to modify matrix materials, such as some derivatives derived from fibronectin, collagen or laminin [26, 28]. The most common one is arginine-glycine-aspartic acid (RGD) sequence, derived from fibronectin. Many reports have demonstrated that the modification of RGD sequence can significantly improve the adhesion [29], spread-shape [30], proliferation [21, 31] and differentiation [32] of cells in biomimetic scaffolds. Furthermore, the effect of RGD on cells is a positive correlation function of its density in many researches. For example, in the intestinal stem cell (ISC) expansion and organoid formation model, RGD can stimulate ISC colony formation in a concentration-dependent pattern [31]. Certainly, the effects of RGD on cell fate are different for different types of cells or different states of the same kind of cells. For example, sequential regulation of macrophage phenotype through dynamic regulation of RGD-patterned surface has been clarified [33, 34]. Most of the researches are focused on a single receptor–ligand, but cellular behavior is often regulated by multiple ECM ligands [35, 36]. Researching each receptor-ligand relationship is beneficial for precise regulation of cell behaviors. The synergistic effect of the combination of RGD and YIGSR peptides on endothelial cell adhesion and spread was demonstrated in 2005 [37]. This is of great significance for many subsequent related studies. Therefore, when considering the modification of cell-adhesion ligands to matrices, it is important to not only consider the effect of single ligands on cells but also synthesize the whole process of cell growth and design rational ligands.

### Mechanical properties

The mechanical properties of the ECM or matrices also have a significant impact on cell behavior. In 1893, Roux W. proposed that mechanical forces could be used to shape tissues and organs during embryonic development [38]. However, it was not until the 20th century, with the advent of biophysical and molecular technologies, that the mechanism by which cells convert mechanical forces into biochemical signals was gradually elucidated [39]. Subsequently, numerous studies have leveraged mechanosignaling pathways to engineer matrix materials for precise cellular regulation. For instance, the YAP/TAZ signaling pathway, which integrates both mechanical and biochemical signals to govern cell behaviors, has been demonstrated to correlate with pro-tumorigenic mechanisms. Recent systematic reviews on YAP/TAZ-based organoid culture systems highlight their dual significance: not only advancing tumor organoid development and expanding their application domains but also deepening our mechanistic understanding of YAP/TAZ dysregulation in cancer progression [40]. The biohybrid hydrogel composed of calcium silicate (CS) nanowires and gelatin methacrylate (GelMA) has been proved to regulate the expression of the mechanical sensory, yes-associated protein (YAP) to stimulate the development and maturation of organoids [41]. As a specialized mechanosensitive receptor, PIEZO channels exhibit the ability to transduce diverse forms of mechanical stimuli into cation influx. Leveraging their nanoscale curvature deformation, PIEZO proteins can detect piconewton-scale forces, becoming activated within milliseconds and subsequently undergoing rapid inactivation. By incorporating these exceptional mechanosensitive properties into the design of matrix materials, it may be possible to precisely regulate cellular behaviors [42]. Here, we will focus on how stiffness and viscoelasticity of matrices regulate biochemical signals to affect cell adhesion, migration, proliferation and differentiation [43] (Figure 1C).

The stiffness of ECM varies from tissue to tissue, which depends on the function of each tissue. Moreover, abnormal stiffness of the ECM has been proved to be a precursor to many diseases (such as atherosclerosis [44], neuroinflammation [45] and cancer [46, 47]). Therefore, the stiffness of the cell culture matrices *in vitro* is a critical factor influencing cell behavior [48]. Collins *et al*. had demonstrated that adding of a very low volume percentage of the stiff microstructures into 3D hydrogels could greatly alter the morphology, clustering and gene expression of human mesenchymal stem cells (MSCs) in 2010 [49]. Subsequently, in 2016, there were studies using hydrogels of different stiffness to induce different types of stem cell differentiation [50]. At the organoid level, studies have demonstrated that stiffness-tunable hydrogels (stiffness ranging from 0.69 kPa to 2.24 kPa) engineered from peptide amphiphiles enhance the formation and proliferation of cerebral organoids, with softer hydrogel formulations exhibiting superior performance in supporting structural maturation and cellular viability [51]. Interestingly, however, when culturing liver organoids in polyisocyanopeptides (PIC) hydrogels, the 12 Pa stiffness formulation more effectively differentiates organoids into hepatocyte-like phenotypes with critical hepatic functions compared to 38 Pa counterparts [52]. The stiffness of ECM not simply affects the normal proliferation and differentiation of cells, soft and normal ECM or passivated cell mechanical conduction have been proved to prevent cell reprogramming into tumor [53]. Many interesting studies on tumor cells show that the high ECM stiffness can promote the transformation of tumor cells into a malignant phenotype and facilitates the invasion and metastasis of tumor cells [54, 55]. For tumor organoid culture, studies comparing pancreatic organoid growth in matrices with stiffnesses of 1.4 kPa, 3.1 kPa, 8.2 kPa and 20.5 kPa revealed that distinct tumor cell signaling pathways are activated under different stiffness conditions. Designing stiffness-tunable organoid matrix materials enables precise modulation of organoid growth and proliferation [56]. The marked biological divergence among cerebral organoids, hepatic organoids and pancreatic ductal adenocarcinoma organoids dictates distinct optimal stiffness values for their matrix materials, highlighting the necessity of tailoring material design to the intrinsic characteristics of each organoid type.

Another remarkable mechanical property of the ECM is viscoelasticity, which means that any deformation of the polymer molecules that adapts to stress when applied by an external force is a function of time and cannot be completed instantaneously [57, 58]. Stress relaxation is defined as the transition of polymer molecules through a series of intermediate states to an equilibrium state adapted to external forces [23, 59]. The half of the time required for this process, known as the stress relaxation half-lives, is frequently used to characterize the viscoelastic properties of materials [21]. Although the role of matrix viscoelasticity in directing cell behavior is not fully understood, some studies have preliminarily proved that the effects may be attributed to the regulation of corresponding signaling pathways [16, 60–62] (Figure 1D). Some matrix materials that mimic the viscoelasticity of natural ECM have been shown to have good ability to induce cell migration, proliferation and differentiation during 3D culture *in vitro*. Among them, the effect of stress relaxation on MSCs are very attractive. The stress-relaxation half-lives is related to the self-renewal or quiescence of MSCs [63]. Moreover, fast stress relaxation can also promote the osteogenic differentiation of MSCs by activating transient receptor potential vanilloid ion channel [17] (Figure 1E). For instance, alginate hydrogels with higher viscosity (70 and 48 kDa hydrogels are more viscous than 170 kDa hydrogels) suppress tissue formation while promoting bovine chondrocyte proliferation to generate cartilage organoids [64]. Similarly, the viscoelastic properties of collagen-nanocellulose hydrogels are proven to be a determinant for intestinal organoid formation and development [65]. Dynamic DNA-crosslinked matrices, due to their superior viscoelasticity, where stress-relaxation times can be tuned across four orders of magnitude to recapitulate the mechanical characteristics of living tissues, have garnered attention in organoid culture [66].

In conclusion, it is essential to regulate the stiffness and viscoelasticity of the matrix with precision when constructing and studying organoids. These parameters are crucial for accurately simulating and applying organoids in a realistic manner.

### Matrix geometry

The geometric structure of hydrogels can be simply divided into two categories: porous structure and fiber network structure, which is similar to natural ECM. The porous structured materials can also be further divided according to pore size: nanoporous (mean pore size ∼5 nm) and macroporous (mean pore size ∼120 μm) [67]. Because the geometry of hydrogels is always closely related to ligand density and material physical properties, it is challenging to explore the structure-induced changes in cell behavior. So far, however, there has been some enlightening research findings on the effects of matrix geometry on cell behavior.

For porous structured hydrogels, Nih *et al*. [68] have demonstrated that porous hydrogels are more favorable than nonporous ones for neural progenitor cells (NPCs) migration to the lesion and can effectively reduce gliosis and inflammation. However, most research in this field has focused on the effect of the pore size. In 2012, Shepard *et al*. [69] proposed that the macropore structured hydrogels could enhance cell infiltration, transduction and influences tissue development. Then, in 2017, Fu *et al*. [70] showed that endothelial cells directly encapsulated in large pore structured hydrogels exhibited the better angiogenic effect. In addition, for MSCs large pore size hydrogels promoted osteogenic differentiation, while small pore (<125 μm in diameter) size was conducive to maintain stemness and undifferentiated [18] (Figure 1F).

For fiber network structured hydrogels, the arrangement and compactness of fibrin are the key factors affecting cells. For example, MSCs cultured in 3D within peptide nanofiber scaffolds can successfully differentiate into chondrocytes [71]. Moreover, uniaxially aligned nanofiber network is a core factor affecting the nerve regeneration [72]; the direction of nanofiber can guide the growth direction of neurites [73]; and the diameter of fiber can influence proliferation and differentiation of rat-hippocampus-derived adult neural stem cells [74]. One study has shown that fiber gel is more conducive to the proliferation and differentiation of bone marrow mesenchymal stem cells than porous gel [75]. However, this phenomenon has been attributed to enhanced Yes-associated protein activity by extending the punctate adhesion and alignment of actin filaments rather than the fiber network of the matrix [19] (Figure 1G).

## Matrix material

As the influencing factors of cell–matrix interactions are being explored, a growing number of well-defined hydrogel materials have been engineered for 3D cell culture. From biopolymer derived materials to semi- or fully synthetic materials with fully tunable mechanical and chemical properties, the common features of these materials are cell–matrix adhesion ability, ECM-like mechanical properties and structures, enzyme sensitivity, etc. In this section, we outlined the relevant uses of biopolymer hydrogels. Then, we report the application of fully synthetic polymer and biohybrid hydrogel systems, ending with a summary of some advances in protein-engineered hydrogels work (Table 1). This classification system exhibits incomplete coverage of organoid culture matrix material categories, primarily due to insufficient extant research on alternative material classifications within the current scientific literature, thereby precluding their incorporation into the present analytical framework.

**Table 1.** Summary of hydrogels for organoids

Materials class	Material	Advantages	Application	References
Natural biopolymer materials	Matrigel	Universality	Suitable for the cultivation of almost all types of organoids	[13]
	Collagen	ECM-like property	PDAC organoids; microscopic cystic organoids	[76]
	Gelatin	Biodegradable; cheap; rich in RGD; cell adhesiveness	CRC organoids	[77]
	GelMA	Photoinduced radical polymerization; tunable mechanical properties	Organizational regenerative medicine	[78]
	HA	Chemical modification versatility; Specific identification of tumor biomarker	Breast cancer organoids	[79]
Synthetic polymer materials	PEG	Deformable backbone; modifiable terminal groups	Normal and cancerous pancreatic organoids; intestinal organoids	[56]
	PIC	Thermoreversible; viscoelasticity	Liver organoids	[52]
Biohybrid polymer materials	oxime-crosslinked HA hydrogel	Cultured organoids are closest to real breast cancer	Breast cancer organoids	[80]
	TG-PEG/HA	Tunable physical and biological properties	Bone Marrow Organoids	[81]
	Alginate-PEG hydrogel	Tunable stress relaxation rate	3D culture of MSCs	[32]
	PPTase-cross-linked semisynthetic hydrogel	Faster cross-linking; promising biological characteristics	3D cell culture in vitro	[82]
	starPEG-heparin hydrogel	Precisely tuned polymer network properties	3D embedded human vascular endothelial cell culture in vitro	[83]
Protein- engineered hydrogels	hyaluronan elastin-like protein	independently quantitative specification of stiffness, stress relaxation rate and integrin ligand concentration	Promote the proliferation and differentiation of patient derived cells to form wavy lumens	[84]

### Natural biopolymer materials

Matrigel is the most used matrix for organoid culture which consists mainly of laminin, collagen IV, nidogen, proteoglycans and several growth factors [13]. However, due to its well-known drawbacks and the urgent need to well-defined matrix materials with low immunogenicity, various materials have been explored as alternatives. Combined with the common characteristics of Matrigel and ECM, the hydrogels composed of biopolymers and their combinations, including collagen, gelatin and hyaluronic acid, are considered to have great potentiality.

Collagen is not only abundant in normal tissues and organs but also an important component of tumor microenvironment [85], for example, collagen rich condition may trigger local hypoxia [76, 86]; for pancreatic ductal adenocarcinoma (PDAC), remodeling the collagen-rich ECM by regulating signaling pathways can inhibit malignancy of cancer cells and break drug delivery barriers [87, 88]. The effect of collagen on tumor development has been a hot spot of research for long time. But from another perspective, collagen-based hydrogels would be an excellent choice for building 3D models of tumors and organoid *in vitro*. For example, the research on 3D collagen matrix which designed for human umbilical vein endothelial cells adhesion, diffusion and proliferation had been completed [89]. Khodayari *et al*., who had investigated the ability of collagen I hydrogel to promote the construction of *in vitro* 3D heart model, found that the obtained organoids have higher angiogenesis ability and show promising therapeutic potential for cardiovascular disease [90]. Then in 2017, Sachs *et al*. [91] extended microscopic cystic organoids self-organized by Lgr5^+^ stem cells to the next macroscopic tube formation with the help of the collagen hydrogel. Recently, it was pointed out that the mechanical properties of collagen will determine the fate of cells encapsuled inside it [92, 93]. It is believed that these mechanisms will further guide the design of advanced materials.

**Figure 2.**
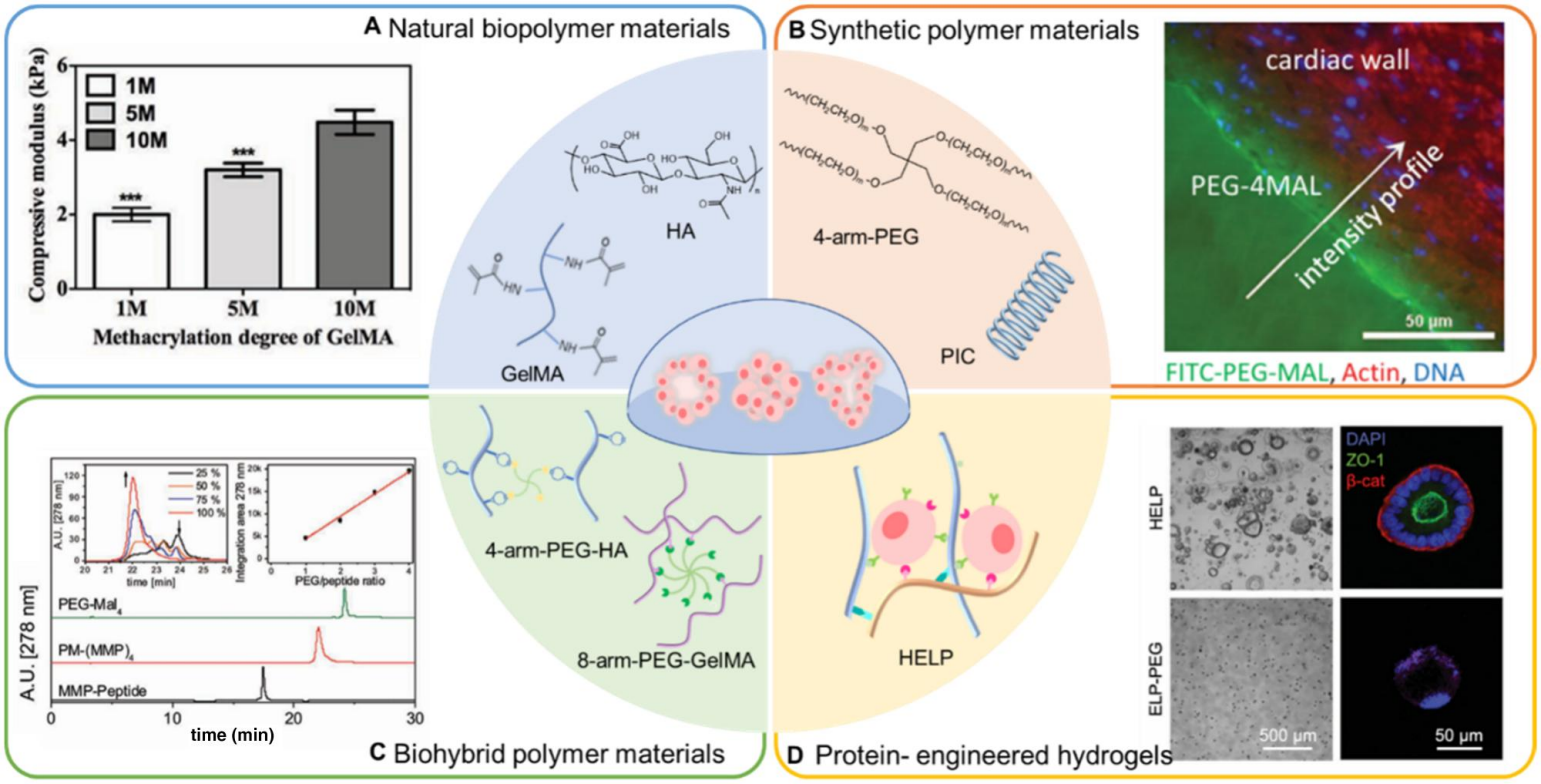
Classification of hydrogel materials for organoid culture. (**A**) Natural biopolymer materials, such as HA and GelMA. Compressive modulus of GelMA hydrogels with different methacrylation degrees [98]. (**B**) Synthetic polymer materials, such as 4-arm-PEG and PIC. Fluorescence intensity profiles for FITC-PEG-MAL illustrate a physical incorporation depth of hydrogel into tissue of approximately 50 μm [99]. (**C**) Biohybrid polymer materials. HPLC chromatogram of purified PEG-(MMP)_4_ conjugates compared to the precursors MMP-peptide and PEG-(maleimide)_4_ conjugates [83]. (**D**) Protein-engineered hydrogels. Brightfield and confocal micrographs at 6 days post-seeding of organoids grown in HELP and ELP-PEG matrices [84].

Gelatin, obtained via the irreversible denaturation of collagen protein, is a well-known biodegradable and biocompatible material [94, 95]. In this work by Van Den Bulcke *et al*. subjected methacrylate gelatin (GelMA), which contain most methacrylamide groups and a few methacrylate groups and can undergo photoinduced radical polymerization. Since the first synthesis report [96], the physical and biochemical properties of GelMA have been intensively studied from tissue engineering to drug and gene delivery. And because it retains many advantages of gelatin such as high biocompatibility, degradability, cell adhesiveness and has excellent tunable mechanical properties [97], GelMA is more widely used than gelatin (Figure 2A). In recent years, GelMA has shown outstanding performance in bone tissue engineering and angiogenesis. For example, a 3D composite GelMA hydrogel scaffold was designed to promote the osteoblastic differentiation of human dental pulp stem cells and achieve simultaneous synergistic osteogenesis of multiple sites by Liang *et al*. [100]; GelMA 3D printing ink can be used in the preparation of breast adipose tissue restoration materials [101]; GelMA microsphere can be used to manufacture hollow organoids by coaxial parallel flow capillary microfluidic device [102].

Unlike collagen and gelatin, HA is a glycosaminoglycan that is widely found in mammalian ECM. Because of the excellent intrinsic biocompatibility and chemical modification versatility, HA are highly attractive for various technologies such as 3D cell culture, bioprinting and tissue repair. It has been reported that 3D printed HA hydrogel can imitate natural ECM, which is a promising material to maintain cell vitality and promote soft tissue repair [103]. And HA is considered as one of the important participants in cancer development and upregulated in many cancers [104]. Furthermore, recent studies have shown that HA/CD44 interactions have diverse roles in different cancer stages [105]. These advantages above make HA an ideal material for culturing breast cancer cells *in vitro*. In 2019, Baker *et al*. designed a novel oxime-crosslinked HA hydrogel which has the capacity to grow breast cancer spheres in 3D [80]. And compared to the cell obtained in Matrigel, the breast cancer cells grown in the new HA hydrogel are the most similar to the orthotopic xenografts. Upon excitation, they further improved this HA hydrogel platform by introducing matrix metalloproteinase-cleavable crosslinker, extending it to construct nine different cancer types *in vitro* [106].

### Synthetic polymer materials

Not just biopolymers, many synthetic polymers, designed with similar properties of ECM, also exhibit promising ability to support cell growth. As presented above, an increasing number of studies have shown that not only the adhesive properties but also the mechanical properties and geometric configuration of the ECM are critical for organoid growth, which reveal the need for a highly adjustable matrix. Biochemical and biophysical properties can be designed on demand and their biosafety can be systematically tested, allowing for the wide use of synthetic polymers. For instance, polyethylene glycol (PEG) is a promising synthetic productive matrix with deformable backbone and modifiable terminal groups, supporting expansion of murine and human organoids [107], such as intestinal [31, 108, 109], lung [110], liver [111] and pancreatic ductal [56].

At present, multi-arm PEG is widely used in research. The terminal groups of PEG are modified by different molecules for specific purposes, and the commonly used cross-linkers are protease-degradable peptides. Some protein additives, such as RGD, laminin and collagen, may also be added into the system to promote bioactivity. An 8-arm PEG activated by vinyl sulfone cross-linked via peptides sensitive to matrix metalloproteinase (MMP) was reported to support the growth of normal and cancerous pancreatic organoids [56]. Moreover, the FN-mimetic peptide PHSRN-K-RGD, the GFOGER peptide and the BM-binding peptide are used as protein additives to facilitate cell adhesion and broader retention of matricellular proteins secreted by cells, and tests indicate that the three protein additives had complementary effects. Notably, the laminin–integrin interactions are demonstrated to play a functionally significant role in pancreatic cancer organoids. Moreover, modular design strategies are also utterly common in multi-arm PEG. A modular synthetic hydrogel has been designed to tune hydrogel stiffness to meet the demand for matrix stiffness at different stages of intestinal organoid development [31, 109]. The regulation was achieved by adjusting the ratio of the two units, modified by vinyl sulfone or acrylate to the 8-arm PEG terminal groups, respectively. Maleimide, due to its rapid reaction kinetics, mild reaction conditions and the ability to facilitate cross-linking, is also widely used to modify multi-arm PEG [99] (Figure 2B). Experiments have shown that 4-arm PEG-maleimide (PEG-4MAL) exhibits a wider range of Young’s moduli, higher encapsulated cell viability and easier bioligand incorporation than hydrogels based on 4-arm PEG-acrylate, 4-arm PEG-VS and UV photo-cross-linked PEG-diacrylate. Synthetic platforms based on PEG-4MAL show a striking capacity in ascertaining the regulation of epithelial morphogenesis [112] and promoting the formation of human intestinal organoid from pluripotent stem cells [108, 110].

Although various PEG-based platforms have demonstrated enormous latent capacity in organoid culture, their gelation process requires the introduction of cross-linking agents or enzymes, which limits their applications. Thermoreversible hydrogel may be an alternative to chemically or enzymatically cross-linked hydrogels, such as polyisocyanopeptides (PIC). PIC is an easily modified bioinert material, which would gel below 16°C with minutes [113]. Hydrogels based on PIC have been proved to possess the viscoelasticity and sensitive chemical and mechanical signal sensing capability meaning that they are suitable ECM mimics [114]. A study showed that PIC-based hydrogels optimized by hrlaminin-111 own excellent ability to support the long-term expansion and differentiation of liver organoids [52]. The proliferation or differentiation environment is regulated by adjusting the stiffness of hydrogel. The thermos-responsive properties of PIC hydrogels not only are advantageous for *in vivo* applications but also make them very convenient for the retrieval of organoids from hydrogels, which is profit for downstream information acquisition. Waterborne biodegradable polyurethane (WBPU) hydrogel scaffolds also show the potential for organoid culture. The 3D lung cancer model established by the WBPU hydrogel scaffold exhibit similar protein expression to that of *in vivo* tumors, and it also closely resembles *in vivo* tumors in terms of resistance and tolerance to nanoparticle drugs, potentially providing valuable research data for clinical trials [115].

Due to the tunable biochemical and biophysical properties, many high-throughput studies have been conducted based on synthesis hydrogels. The related content will be introduced in detail in section downstream information readouts.

### Biohybrid polymer materials

Above we introduced biopolymer and synthetic polymer materials, and we can easily find that their advantages are complementary. Therefore, biohybrid polymer may be a feasible solution. Common polymers used for organoid culture applications include PEG, peptide, HA and GelMA.

Several biohybrid hydrogels based on PEG have been developed for organoids cultured *in vitro*. PEG is so widely used because the end group can easily react with another polymer to cross-link, thus, forming an interpenetrating network. A novel oxime-crosslinked HA hydrogel as mentioned above, used a modular design approach. Fast-reacting HA-aldehyde and slow-reacting HA-ketone were combined with PEG-oxyamine via oxime click chemistry to grow breast cancer organoids *in vitro* [80]. In another study, the transglutaminase (TG) factor XIII was used as enzymatical crosslinker to incorporate PEG and HA [81]. Tunable physical and biological properties are shown to be feasible for bone marrow organoid formation at different stages. As combining features of PEG and HA, the TG-PEG/HA hybrid hydrogels possess high resistance to enzymatic degradation of PEG and low immunogenicity of HA, exhibiting stronger hematopoietic bone marrow stromal cells and hematopoietic stem and progenitor cells maintenance and proliferation capability than single kind hydrogel. Not only the HA, alginate is also common in forming hybrid hydrogels with PEG. As mentioned in section mechanical properties, different mechanical properties of the surrounding would induce different respond of cells, such as spreading, proliferation and differentiation. Original work by Chaudhuri *et al*. [21] have showed a novel alginate/PEG hydrogel, which can be tuned the stress relaxation rate independently of initial elastic modulus, polymer concentration, degradation and RGD cell-adhesion-ligand density. The concept of stress relaxation-dependent cell behaviors such as spreading, proliferation, differentiation have been confirmed in MSCs.

Another major natural biopolymer materials used for the generation of biohybrid hydrogels with PEG is peptide. Adhesive peptide ligands or degradable peptide cross linkers were used for a long time when designing peptide-based biohybrid hydrogels, such as RGD sequence [116] and matrix metalloproteinase cleavable sequence [30, 106]. Or by using materials that are inherently degradable, for instance, a photodegradable PEG-based hydrogel [117], which are synthesized by using PEG and GelMA, can degrade via UV irradiation due to the photo-responsive property of GelMA. The phosphopantetheinyl transferase (PPTase) catalyzed formation of hybrid hydrogel was first reported by Mosiewicz *et al*. [82] in 2010. PPTase mediated cross-linking is covalent, rapid and highly specific and PPTase has a small size, high expression rate and is easy to purify, making PPTase cross-linking an ideal target for biomaterial engineering. Mild and highly selective crosslinking chemical reactions also have received particular attention, such as Michael type addition reactions. However, this reaction only applies to water-soluble components. Therefore, various strategies have been developed to break this limitation. Tsurkan *et al*. [83] reported a starPEG-peptide conjugated with terminal thiol groups with precisely tuned polymer network properties, which enabled to introduce a water-insoluble peptide into the hydrophilic hydrogel environment (Figure 2C).

Of note, the design of cell-instructive biohybrid hydrogels is not limited to the PEG or the incorporation of natural biopolymer and synthetic polymer. For example, a gelatin-HA hybrid hydrogel crosslinked by enzyme with high matrix stiffness had been proved to facilitate Colorectal cancer(CRC) patient-derived tumor organoids (PDOs) growth and metabolism *in vitro* and support the drug screening of various CRC therapeutic drugs [77]. Common polymers for organoid culture applications also include β-cyclodextrin and adamantane [118], PIC [52, 116] and polyacrylamide [119].

Consequently, through the rational design of the chemical composition and physical properties of biohybrid polymers, it is possible to simulate the microenvironment of human tissues, thereby promoting cell proliferation and differentiation. These materials can be employed not only for the cultivation of artificial organs but also play an important role in the fields of regenerative medicine and tissue engineering.

### Protein-engineered hydrogels

Protein-engineered ECM-mimetics offer great opportunities to address the challenge of harnessing specific ECM features to regulate tissue morphogenetic regulation, as the predictable biofunctionality and precise tunability of such biomaterials can allow for an independent control over their cell-instructive characteristics [120]. Proteins-engineered hydrogels, like biohybrid hydrogels, are also considered to combine the advantages of natural and synthetic materials due to the modularity, tunability and sequence specificity [121]. Developing 3D materials that simultaneously support proliferation and stemness maintenance of NPCs is of great interest to expand the clinical applications of stem cells. Therefore, Madl *et al*. [15] investigated the effects of matrix stiffness and degradability on the stemness maintenance of NPCs using 3D protein-engineered hydrogel. Moreover, they identified that the matrix remodeling, which can facilitate cell spreading, allow cell–cell contact and initiate downstream β- Catenin signaling, is essential for the stemness maintenance of NPCs in protein-engineered hydrogels. The similar strategy, which designed a hyaluronan elastin-like protein (HELP) hydrogel [84], has been employed to study the human patient-derived intestinal organoids culture. The HELP allows independently quantitative specification of stiffness, stress relaxation rate and integrin ligand concentration. HELP not only enabled proliferation and differentiation of patient derived cells to form undulating lumens, but also maintained a stem-like quality for up to 12 passages, suggesting the clinical translation potentiality of tailorable materials (Figure 2D).

### DNA-crosslinked matrix (DyNAtrix)

The DNA hydrogel (DyNAtrix) is a dynamically crosslinked matrix engineered through DNA nanotechnology. By tuning DNA sequences, it enables systematic modulation of stress-relaxation times to precisely mimic the mechanical properties of living tissues, providing cells with dynamic mechanical cues. The hydrogel demonstrates reversible liquefaction under mechanical stress and rapid self-healing. These advantages confer DyNAtrix with superior organoid-culturing capabilities. Studies by Peng *et al*. reveal that DyNAtrix supports high viability, proliferation and morphogenesis in human mesenchymal stromal cells, pluripotent stem cells, canine renal cysts and human trophoblast organoids [66]. Notably, DyNAtrix can be combined with 3D printing technology, which is expected to accelerate the progress of organoid research.

## Applications of organoids in biomedicine

With the development of various hydrogel platforms based on human organoids, the goal of building almost physiological and self-renewing organoid model systems is gradually approaching. In fact, currently 3D organoids have shown striking similarities to natural organs in terms of gene and protein expression, metabolic function and microstructure. With ongoing efforts to develop organoid platforms, the pathogenesis and diagnostics of various human diseases have been modeled. And these platforms also provide the possibility for specific disease tissues or organs to be used in drug testing and precision medicine applications (Table 2). Here, based on the applications of organoids in the biomedical field, we focus our discussion on three key directions: cancer oncogenesis and progression research, drug screening and personalized medicine, while also exploring future prospects for organoid hydrogel development.

**Table 2.** Summary of the construction and application of different types of organoids

Disease type	Source of organoids	Culture system	Application	References
Colorectal cancer (CRC)	CRC patients	Basal floating culture medium containing 2% Matrigel (Corning).	Drug screening; colorectal tumor organoid library	[122]
CRC	CRC-PDX tumors	Enzymatically crosslinkable, ECM-derived, gelatin-Ph, HA-Ph, gelatin-Ph/HA-Ph hydrogels.	Drug screening; exploring the characteristics of matrix materials that affect the metabolic growth of organoids	[77]
CRC	CRC patients	Matrigel (Corning) or BME R1 (Trevigen)	Biological mechanisms and pharmacological interventions of colorectal cancer	[123]
CRC	CRC biopsies	Matrigel	Drug screening	[124]
Pancreatic acinar and ductal carcinoma	Human stem cell (Hues-8 cells)	Matrigel	Modeling exocrine development and diseases; demonstrating lineage tropism and plasticity for oncogene action in the human pancreas	[125]
Pancreatic ductal adenocarcinoma	PDAC patients	70% BME	Drug screening; emphasizing the importance of personalized approaches for effective cancer treatment	[126]
PDAC	PDAC patients	PDMS devices cast using standard soft lithography programs	Facilitate treatment decisions for personalized therapy	[127]
Liver cancer	Human liver tumor specimens	BME	Drug screening; biomarker identification	[128]
HCC and CC	Human HCC specimens	Matrigel	Exploring the relationship between LGR5 marks with tumor initiation and tumor drug resistance	[129]
Liver cancer	Reprogrammed human hepatocytes (hiHeps)	Ultra-low attachment plate (round bottom type; Elplasia); hepatocyte-maintaining medium (HMM)	A model of initial alterations in human liver cancers	[130]
Kidney disease	iPSCs	Matrigel	Drug screening	[131]
Upper tract urothelial carcinomas (UTUCs)	UTUC patients	Matrigel	Drug screening; elucidate UTUC pathophysiology	[132]
Endometrial disease	Biopsies	70% Matrigel	Drug screening; capture endometrial disease diversity	[133]
TNBC	Trp53-null mammary tumor cell	Matrigel	Drug screening; exploration of the mechanism of epithelial mesenchymal transition (EMT)	[134]
TNBC	Patient tumor samples	Matrigel	Drug screening	[135]
TNBC	Patient tumor samples	Matrigel	Drug screening; precision medicine	[135]
Cardiovascular disease	50% hiPSC-CMs and 50% non-myocyte	Non-adhesive agarose hydrogel molds	Drug screening; model diseases with non-genetic pathological factors	[136]
Gastrointestinal diseases	Stem cell	Hydrogel-based U-bottom microwell arrays	High-content phenotypic drug screening;	[137]
Pan-cancer	Patient tumor samples	Matrigel	Precision medicine	[138]
Glioblastoma	Patient tumor samples	Matrigel	High-throughput drug screening; modeling primary human glioblastoma *ex vivo*	[139]

### Organoids in cancer pathogenesis research

**Figure 3.**
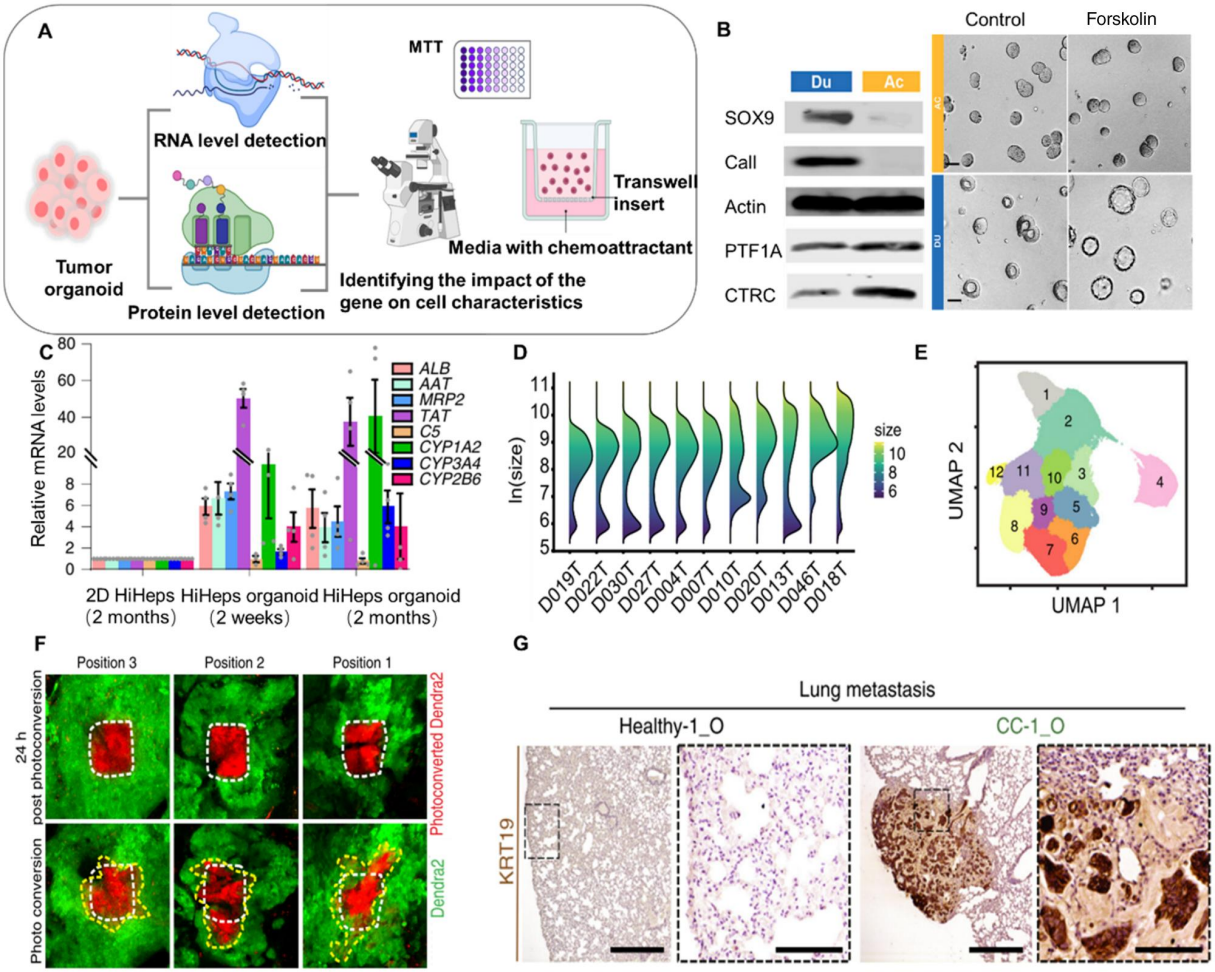
Organoids for cancer oncogenesis and progression research. (**A**) Schematic of organoids in cancer oncogenesis and progression research. (**B**) Immunoblot analysis for acinar and ductal markers. The numbers under the blots represent normalized signals of protein bands (left). Morphological changes (right) of day 8 DUs (blue) and ACs (orange) in response to 10 mM forskolin treatment (2-h incubation) [125]. (**C**) RT–qPCR data of hepatic genes in long-term cultured 2D hiHeps and hiHep organoids [130]. (**D**) Organoid size distribution across organoid lines. (**E**) UMAP representation of DMSO treated and drug treated organoids. Graph-based clustering of organoids by morphology with 12 resulting clusters [123]. (**F**) Representative intravital images of orthotopic murine AKP Dendra2-reporter intestinal tumors. (down) Photoswitched areas at time point 0 h. (up) Same imaging microenvironments 24 h after photoconversion. Green represents nonconverted Dendra2, whereas red highlights the photoconverted Dendra2 tumor cells. White dashed lines highlight the photoswitched areas at beginning of the experiment. Yellow dashed lines mark the edges of the red Dendra2 areas 24 h after photoconversion. Scale bar, 100 µm [145]. (**G)** Lung metastases derived from CC-1 tumoroids transplanted under the kidney capsule (right) were identified using an antibody. No metastases were found in the lungs of mice injected with healthy-1 organoids (left) [128].

Cancer is a major health problem worldwide. Developing effective treatment is one of the ways to further improve the quality of life and prolong survival of cancer patients. However, traditional cancer models, such as 2D cancer cell lines, genetically engineered mouse models and primary patient-derived xenograft (PDXs), have poor reproducibility for human tumors, which has led to unsatisfactory results in clinical trials of drugs that work well in traditional cancer models [140, 141]. The failure of these traditional models may be attributed to treating tumors as random mixtures of cells and ECM, while tumor rather resemble organs [142]. The formation of tumor is a complex process that is often accompanied by the recruitment of fibroblasts, remolding of ECM, establishment of vascular networks and complex interactions with immune cells [143]. Tumor organoids are considered more effective and accurate cancer modeling strategies because they can retain pathological and genetic characteristics of the tissue and respond to treatment [144]. Currently, the fidelity and reproducibility of various tumor organoids are constantly improving, which will further promote *in vitro* research on cancer oncogenesis and progression (Figure 3A). Reproducing the mechanical properties and cellular diversity of the TME is an important step to establish a reliable tumor model *in vitro*. TMEs constantly evolve with tumor growth and is a complex environment that consist mainly of immune cells, stromal cells, blood vessels, nutrients, growth factors and extracellular matrix [146]. Recently, Lee *et al*. [147] reported a high-throughput oil-in-water droplet microtechnology, which uses collagen and Matrigel mixed hydrogel together. This technology is applicable to a variety of cancer cells, and can facilitate to establish organoids with microenvironment structures that mimic basement membranes and matrix barriers. The bilayered architecture of tumor organoids allowed the simultaneous assessment of the proliferative and invasive properties of cancer cells. If this technology can be popularized and combined with other types of hydrogel materials, it is believed that it can further narrow the gap between preclinical cancer research and clinical patient results. In conclusion, recapitulating the biomechanical and cellular diversity of the TME represents a pivotal advancement in developing new-type organoid hydrogels and culturing physiologically relevant tumor models.

High-fidelity tumor organoids serve as the foundation for studying tumor oncogenesis and progression, enabling the acquisition of rich biological information. Huang *et al*. utilized pancreatic acinar/ductal dual-lineage organoid models to systematically dissect the spatiotemporal expression patterns of key oncogenes (KRAS^G12D^ and GNAS^R201C^) during pancreatic ductal adenocarcinoma (PDAC) progression (Figure 3B), revealing the synergistic effects of distinct driver genes in remodeling the tumor microenvironment [125]. Further studies demonstrated through reprogrammed human hepatocyte (hiHep)-derived organoid models that c-Myc overexpression significantly promotes hepatocellular carcinoma progression by regulating stemness maintenance pathways (Figure 3C) [130]. In colorectal cancer (CRC) metastasis research, patient-derived paired primary-metastatic organoid models provide critical tools for unraveling tumor evolution. Betge *et al*. [123] revealed that metastatic organoids exhibit more aggressive phenotypes and metastatic capabilities, with morphological features (e.g. 3D structural dynamics and size heterogeneity) directly reflecting drug response mechanisms (Figure 3D and E). Moreover, an orthotopic approach based on CRC organoids had enabled the visual real-time study of tumor cell dynamics [145] (Figure 3F).Notably, breakthroughs in organoid technology are also reflected in enhanced multi-cancer modeling capabilities. Broutier *et al*. [128] developed a near-physiological culture system enabling co-cultivation of healthy hepatocytes with three hepatocellular carcinoma subtypes, with xenograft assays confirming that primary liver cancer (PLC) organoids retain multidimensional tumor characteristics, including histopathological architecture, genomic profiles and transcriptomic features (Figure 3G). These advances collectively establish an integrated research framework spanning metastasis mechanism elucidation, driver gene functional validation and preclinical drug efficacy evaluation, providing robust technical support for precision.

### Organoids for drug development

**Figure 4.**
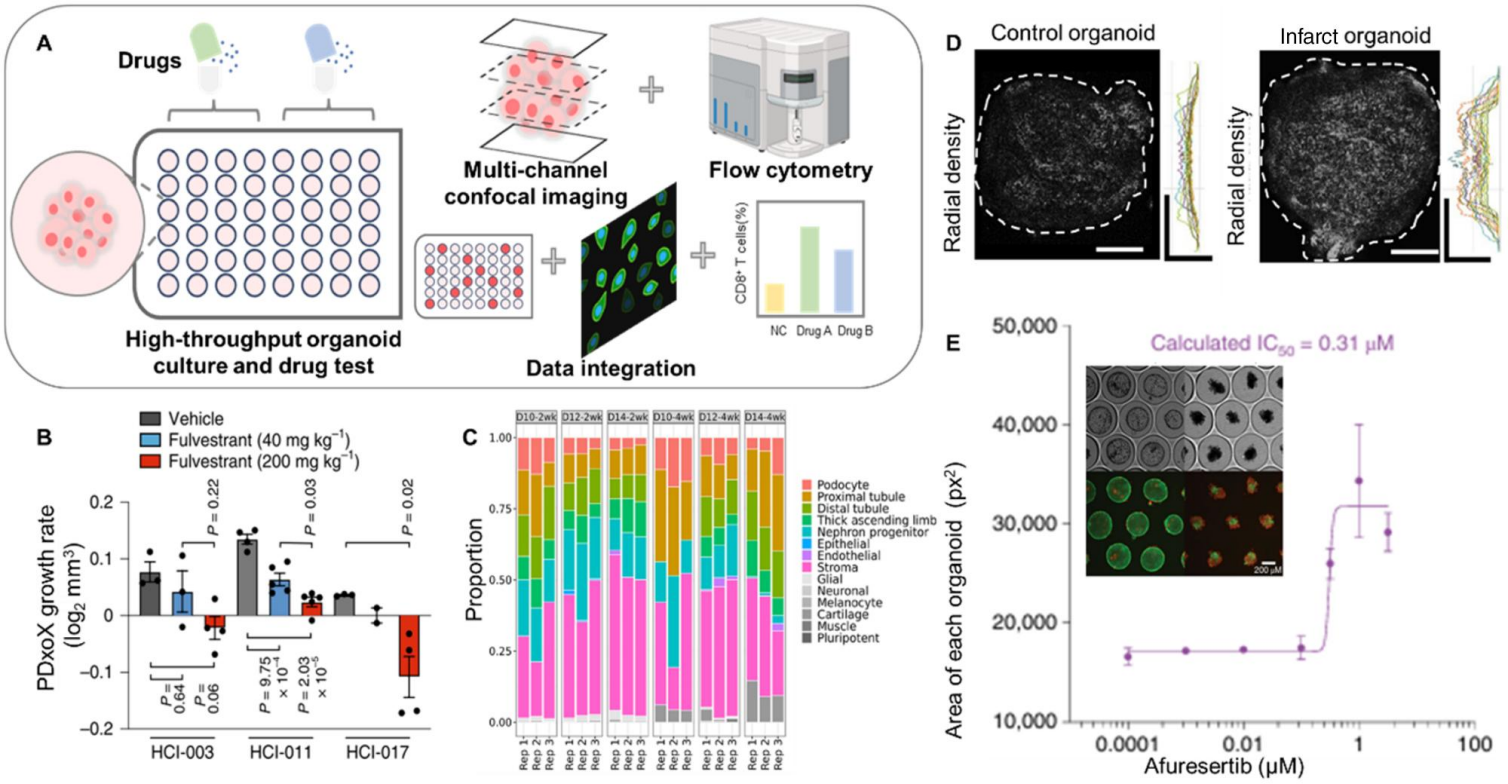
Organoids for drug development. (**A**) Schematic of tumor organoids for drug development. (**B**) HCI-003, HCI-011 and HCI-017 PDxoX tumor growth rate when response to 40 mg kg^−1^ or 200 mg kg^−1^ fulvestrant treatment [135]. (**C**) Stacked bar graph showing cell type proportions of *in vivo* maturation across six time points [131]. (**D**) Confocal z-slice images (>30 µm below the surface of the organoid) of hypoxia-activated image-iT green hypoxia live-cell stain at D10 with radial-density-profile plots of normalized integrated intensities, indicating decreased oxygen (brighter) towards the interior of infarct organoids [136]. (**E**) Representative wide-field and fluorescence microscopy images of the effect of afuresertib on CRC organoids at various concentrations [137].

The traditional preclinical model of the pharmaceutical industry are animal models and 2D cell cultures [141]. However, drug testing based organoid, including drug efficacy testing, toxicology testing, pharmacokinetic testing and drug resistance testing, is considered to be of great potential due to the higher consistent structure and gene level of organoid with human tissues [148]. Organoids designed to mimic pathological tissue can also retain pathological and genetic characteristics of the tissue and respond to treatment[144], which further narrow the gap between animal models and human based clinical trials [149] (Figure 4A). For example, Guillen *et al*. [135] reported a series of matched PDXs and PDX-derived organoids, facilitating the discovery of a potentially effective Food and Drug Administration approved drug for the treatment of triple-negative breast cancer (TNBC). And in the above model, 4-day drug response is the best way to identify drugs with cytotoxicity (Figure 4B). Pharmacodynamics (PD) and pharmacokinetics (PK) are important components of drug research. Recently, human kidney organoids have been used to perform PD and PK studies with GFB-887 (a kind of transient receptor potential canonical 5 inhibitor), determining the therapeutic concentrations of GFB-887 (oral administration) [131] (Figure 4C).Organoids that mimic pathological tissue can also be used to establish biobanks, recapitulating the spectrum of genetic changes underlying disease, allowing the detection of gene drug associations, enabling high-throughput drug monitoring [150].

In addition to cancer, there are many other diseases that require attention. For instance, the cardiovascular disease is also a leading cause of death worldwide [151]. Richards *et al*. [136] reported a cardiac organoid with oxygen-diffusion gradient, which mimic the human heart after myocardial infarction at the transcriptomic, structural and functional levels (Figure 4D). The organoid provided a tissue level model to assess drug-induced/exacerbated cardiotoxicity, including cardiac and fibrotic effects. Endometrial diseases are a major burden of gynecology [152]. A study [153] that designed an endometrial organoid to investigate the mechano-sensitive and chemo-sensitive ion channels, demonstrated that PIEZO1 channels were potential targets for designing endometrial disease-related drugs. Then, they established endometriosis organoids and endometrial cancer organoids to capture the complex information associated with endometrial disease and to provide powerful models for drug screening [133].

Moreover, the application of high-throughput technologies to organoid expansion and *in vitro* culture enables the high-throughput drug screening [154]. Brandenberg *et al*. [137] reported a high-throughput and automated method for organoid culture, which revealed mechanisms of drug action via massive image-based analysis of phenotypes (Figure 4E). And in the above models, the half maximal inhibitory concentrations of drugs are traceable and quantitatively detectable at the organoid level (Figure 4F). A study had encapsulated decellularized extracellular matrix derived from pig liver within highly porous poly(lactic-co-glycolic acid) microspheres (dECM-PLGA PM) utilizing microfluidic techniques for organoid culture. The organoids generated on this platform effectively mimic the drug resistance traits of tumors, offering a sustainable and scalable platform for drug testing [155]. Schuster *et al*. [127] developed a microfluidic platform for dynamic and combination drug screening to test drug effects under different administration conditions. The test process was automatically carried out by the control system, which improved the reliability of drug effect determination and the high throughput design laid the foundation for obtaining statistically relevant quantitative experimental results.

Numerous emerging technologies, such as 3D printing technology [156, 157], had been integrated into organoid drug screening. Thanks to these advancements, organoids are poised to play a significantly greater role in preclinical drug trials. Although the current organoid biobanks that have been established to identify and test new drugs largely focus on cancer, there will be more drug testing organoid models of various diseases and healthy organoids available for toxicology testing in the future.

### Organoids for personalized medicine

**Figure 5.**
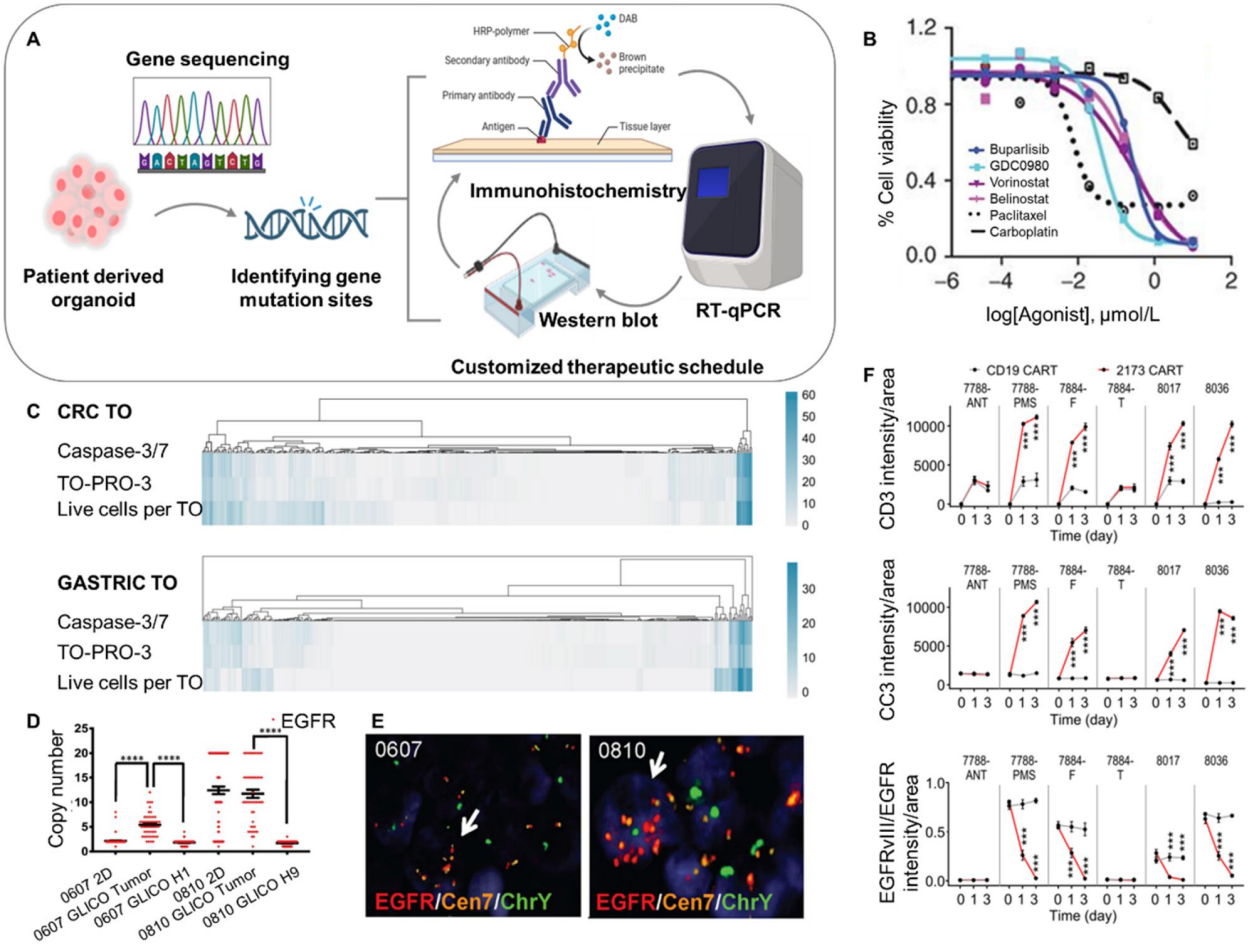
Organoids for personalized medicine. (**A**) Schematic of organoids for personalized medicine. (**B**)The *in vitro* validation of selected drugs in the 3D system [160]. (**C**) Heatmaps show the inverse AUC for all 351 compounds for both the CRC TOs (top) and GASTRIC TOs (down) for each readout [138]. (**D**) Quantification of EGFR copy number variation in 2D or GLICO samples from patients with EGFR amplification. (**E**) Representative images of DNA FISH for EGFR/Cen7/ChrY (Y chromosome) in GLICOs are shown; 0607 GLICOs (from a female patient); 0810 GLICOS (from a male patient) red, EGFR; orange, Cen7; green, ChrY; white arrow indicates tumor cell [139]. (**F**) Summary of quantifications of averaged signal intensity of CD3, CC3 and averaged EGFRvIII/EGFR signal intensity ratio in GBOs after co-culture with either CD19 or 2173BBz CAR-T cells [161].

Personalized medicine, also called precision medicine, refers to a customized medical model based on personal genome information of patients combined with relevant internal environment information such as proteome and metabolome, to design the optimal treatment for the patient in the hope of maximizing the therapeutic effect and minimizing the side effects [158]. Organoids, which are easy to establish and can cover patient genotypes, physiological and pathological changes and other characteristics, are considered to have great potential to assist the development of personalized medicine and optimize current treatment strategies [159] (Figure 5A).

Precision medicine is inseparable from a high understanding of pathogenic genes and patient genomes. Pauli *et al*. [160] combined used whole-exome sequencing (WES), PDO and PDX to assist physician gives the treatment strategy. By sequencing and analyzing genes of patients, this strategy matched the corresponding drug according to their cancer genes. For patients whose genomics has not specified obvious approved targeted therapeutic drugs, *in vitro* drug trials would be conducted to determine effective treatment strategies by using PDO and PDX (Figure 5B). Tumor cases of two uterine malignancies and two CRC were analyzed using this strategy, and optimal combination therapies were tailored for each patient. Larsen *et al*. [138] described a scalable and repeatable high-fidelity pan-cancer PDO culture platform with a high-throughput neural network-based drug analysis systems, which can predict patient specific heterogeneity of drug response. There was a highly significant correlation between the corresponding drug response and the true response for the ten different cancer types tested based on this platform. The authors subsequently evaluated the different active compounds between the two PDO lines using this platform, advancing the application of this platform in precision oncology research (Figure 5C). As previously described, matched PDXs and PDX-derived organoids had been used for precision treatment of ER^+^ breast cancer, demonstrating that the degree of resistance to fulvestrant is different in different patients, and the time of emergence of drug resistance is also different [135]. This model successfully predicted early metastatic recurrence in the liver of a 43-year-old patient with stage IIA TNBC. Then, with drugs selected by using this model, the complete remission of liver metastases in patients lasted for almost 5 months. Recently, harnessing organoid models for precision medicine of glioblastoma has yielded numerous achievements. Linkous *et al*. [139] had successfully constructed a brain organoid glioma (GLICO) model using patient derived glioma stem cells (GSCs) and human embryonic stem cell-derived brain organoids. GLICO could maintain the key genetic characteristics of the parental tumors, and the sensitivity of GSCs to chemotherapy drugs and radiotherapy in GLICO model was also consistent with the *in vitro* test, which greatly encouraged researchers (Figure 5D and E). In another study, by pairing the glioblastoma organoid (GBO) mutation profile with the response to specific drugs, researchers demonstrated that GBOs from different tumors respond heterogeneous to different drug treatments which means that drug efficacy is closely related to the mutation status of the tumor [161]. Their experiments using GBOs to rapidly detect antigen-specific chimeric antigen receptor T cells (CAR-T) were also successful, further demonstrating the implementation of GBOs for precision medicine (Figure 5F). Expanding the library of GBO mutation profiles and corresponding therapeutic drugs may improve the precision of treatment and expand the application scope.

## Challenges

Above, we have discussed the various applications of organoids in the field of medicine. And it is undeniable that the organoid is changing our traditional methods of studying diseases and drugs, helping to bridge the gap between *in vitro* research and medical applications. Although the field of organoids is advancing at an astonishing rate, challenges remain, which can be divided into three aspects, imperfect physiological architecture of organoids, reproducibility and information readouts. Subsequently, we discuss how to address these challenges with the assistance of hydrogels materials.

### Limited level of maturity and lifespan

**Figure 6.**
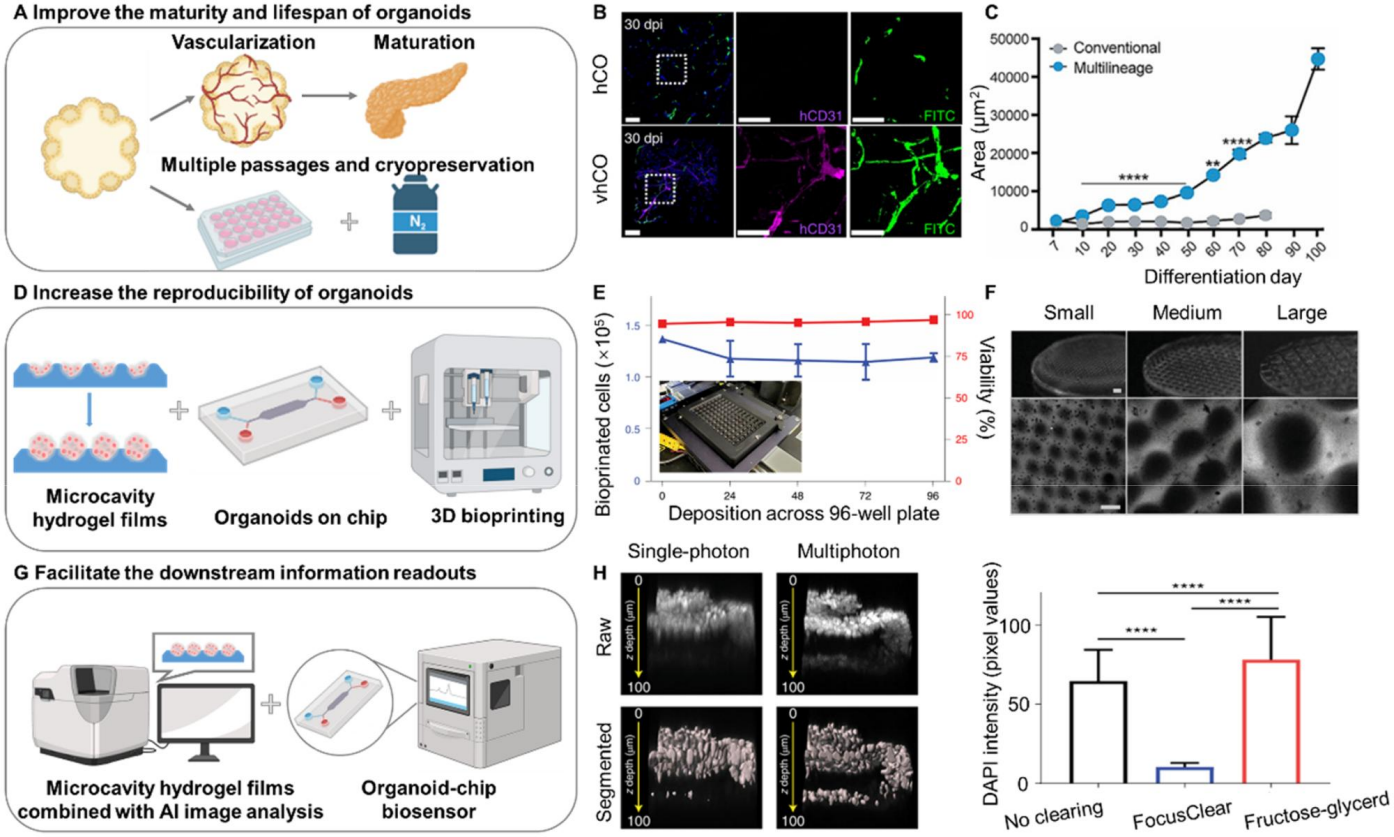
Schematic depiction of next-generation organoids to meet the needs of fundamental and clinical research. (**A**) Improve the maturity and lifespan of organoids. (**B**) Explanted organoids from FITC-perfused mice were stained for human specific [164]. (**C**) Multilineage organoids display an increase in surface area until day 100 of culture [165]. (**D**) Methods of increasing the reproducibility of organoids. (**E**) Quality control assessment of cell number per organoid and viability across a 96-well plate [166]. (**F**) Representative images of microwell HA hydrogels modified with fluorescein and fabricated using silicone molds with different widths and depths [167]. (**G**) Methods of facilitating the downstream information readouts. (**H**) Representative raw images (top) and DAPI-segmented images (bottom) of the same cystic fibrosis organoid imaged with single-photon or multiphoton microscopy (left). Average DAPI intensity with different clearing methods (right) [168].

Currently, no established organoid system can perform all the functions of organs [162], and organoid systems typically only reach the maturation level of fetal tissues [163]. The imperfect physiological structures result in the limited level of maturity and lifespan of organoids, which limits the applications (Figure 6A). An important aspect hampering the maturation of organoids is non-vascularization [169]. Only fully vascularized organoids can deliver nutrients to internal cells and transport metabolic waste from internal cells, ensuring normal growth of internal cells [169]. For kidney organoids, vascularization appears even more important, because the function of the kidney to filter blood and maintain fluid balance is closely related to the complex vascular network [170]. Homan *et al*. [171] remarkably succeeded in producing enhanced vascular abundance kidney organoids on milli-fluidic chips using gelatin by 3D printing technology. Their findings demonstrate that fluidic shear stress is a key environmental cue to promote the vascularization of kidney organoids *in vitro*, which is enhanced under flow. This also suggests that the mechanical properties of the matrix material may be a key clue to breaking through the dilemma of organoid vascularization. Recently, a dECM hydrogel made from fresh porcine kidneys was reported to enhance vascularization and maturation of kidney organoids, probably because dECM can promote the maturation of human umbilical vein endothelial cells and support endothelial cell growth [172]. In addition to gelatin and dECM, composite hydrogels (such as bioinks composed of GelMA and chitin nanocrystals fabricated via 3D printing) can also promote angiogenesis due to the incorporating integrated biomimetic biochemical cues, tunable mechanical properties and three-dimensional mass transport channels [173]. Vascularization also affects oxygen and nutrients supply, which is important at later stages in organoid culture [174]. Cakir *et al*. [164] had presented a method to generate functional vessel like networks that facilitate oxygen and nutrient delivery to the inner-most parts of the organoid, promoting advances in brain organoid (Figure 6B). Moreover, as organoid size increases, the oxygen gradients and insufficient nutrients generated within organoids lead to the death of central cells, which in turn affects the lifespan of the whole organoid [175], thus, establishing large-sized and long-lived organoids is extremely challenging. Advantages such as guaranteeing gas exchange as well as transport of nutrients and waste products have led bioreactors to be considered as efficient routes to extend organoid lifespan and maturity [12, 176]. Silva *et al*. induced the differentiation of human iPSC-derived mesodermal progenitor cells and achieved morphologically multilineage organoids by adding specific growth factors to Matrigel. After continuous culture for 100 days, the average diameter of the organoids reached 2 mm [165] (Figure 6C). But a mere increase in size does not signify true maturity; the cellular composition of organs is complex. For instance, tumors comprise not only cancer cells and vascular structures but also numerous immune cells, which are closely linked to tumor progression and therapy. Thus, cellular complexity serves as a critical criterion for assessing organoid maturity. Studies utilizing hyaluronic acid-gelatin hydrogels to co-culture cancer-associated fibroblasts with colorectal cancer organoids have demonstrated improved preservation of key molecular features of original patient tumors, enabling personalized drug screening a highly valuable advancement in cancer medicine [177].

### Reproducibility

Another challenge of organoid is reproducibility, which will determine the repeatability of the data obtained from it. The scalability of organoid production and the differences in cell composition, structure and function between batches are important factors that affect the use of organoid in high-throughput tests [162] (Figure 6D). Currently, various engineering strategies, such as increasing the degree of automation, the use of well-defined matrices and the control of initial conditions (such as the number of cells seeded), are being used to improve the reproducibility of organoid culture. In a study, by using the cellular extrusion bioprinting approach, Lawlor *et al*. [166] achieved rapid, high-throughput generation of kidney organoids with highly reproducible. The biophysical parameters of organoids (including size, cellular density and spatial conformation) can be precisely controlled via bioprinting and directly fabricated within 96-well plates, significantly streamlining subsequent drug screening. Utilizing this model, researchers quantitatively assessed aminoglycoside relative toxicity through standardized viability and metabolic activity assays, demonstrating the platform's utility in high-content pharmacological profiling (Figure 6E). The synergistic integration of engineered methodologies with matrix hydrogel systems can achieve complementary advantages that surpass the sum of individual components. A representative paradigm involves microarchitected hyaluronic acid (HA) hydrogels, where the authors implemented RGD peptide functionalization to fine-tune the initial storage modulus for enhanced organoid formation efficiency. This approach was further combined with the engineering of micropore templates to achieve spatially controlled self-organization, ultimately yielding uniform-sized alveolar organoids with preserved epithelial polarity, as validated through qRT-PCR analysis of surfactant protein C expression [167]. In this strategy, both microwell size and initial cell seeding density can be accurately regulated, providing a Matrigel free and reproducible method for alveolar organoid culture (Figure 6F).

### Downstream information readouts

Recent advances in bioengineering protocols and microfabrication technologies have significantly improved the standardization of organoid morphology, enabling the production of organoids with uniform size and shape. However, conventional culture systems still face critical limitations in analytical precision due to spatial heterogeneity: organoids distributed across multiple focal planes within standard matrices hinder automated imaging and quantitative data extraction, restricting the implementation of high-content analysis and real-time monitoring strategies [148, 178]. In contrast, micro-engineered hydrogel film platforms address these challenges by confining organoid development to a single focal plane through substrate topography-guided self-organization. This spatial control remains stable throughout prolonged culture periods and repeated medium exchanges. Such technical superiority enables longitudinal tracking of developmental trajectories, from initial cell clustering to mature organoid formation, at both single-organoid resolution and population-level throughput [137] (Figure 6G). Developing methods for evaluating live cells is also crucial. The study had clarified the significance of the concentration and incubation time of resazurin in non-destructive vitality testing in 3D culture [179]. Conversely, fluorescence microscopy is an efficient approach in describing the cellular composition of organoids and the phenotypic similarity of organoids to their original tissue [168] (Figure 6H). These synergistic innovations collectively bridge the gap between organoid generation and actionable quantitative analysis.

Regarding the challenges currently faced by organoids, including limited maturity/lifespan, reproducibility issues and downstream data acquisition, we summarize the following directions for designing organoid hydrogels. To enhance maturity, vascularization strategies such as 3D bioprinting endothelial cells, incorporating decellularized extracellular matrix (dECM) and utilizing bioreactors to simulate fluid shear stress have been employed to improve nutrient delivery and hypoxia mitigation. For reproducibility, automation (e.g. microfluidic droplet printers) and standardized protocols—controlling hydrogel stiffness, ligand density and initial cell numbers—enable scalable, homogeneous organoid production. Downstream readouts are optimized via single-cell RNA sequencing, fluorescence microscopy and engineered platforms (e.g. microcavity arrays) that align organoids in focal planes for high-throughput phenotyping. Additionally, biohybrid hydrogels with tunable stress relaxation and modular designs (e.g. PEG-based systems) mimic dynamic tissue microenvironments, promoting functional maturation. These advances collectively bridge gaps between *in vitro* models and clinical applications, enhancing drug screening accuracy and personalized medicine potential.

## Conclusions and perspective

In this review, we mainly discussed hydrogels for organoid culture. Considering cell-ECM interactions, three aspects of cell-adhesive ligand, mechanical properties, matrix geometry may be key to the design of substrate materials for organoid culture. Strikingly, viscoelasticity has been incorporated into the important mechanical properties of a new generation of organoid culture synthetic matrices, proven to be an important regulator of a range of cellular phenomena [21]. Various synthetic or physically or chemically modified naturally derived materials are currently used for organoid culture in the form of hydrogels. Recently, filamentous phages are, in essence, protein nanoparticles encapsulating the genome, which is considered as a potential unit for preparing hydrogels. Its unique flexibility has attracted a large number of researchers, and existing work has customized filamentous bacteriophages as hydrogel units through genetic engineering or chemical modification in the field of biomedicine [180]. We are confident that flexible filamentous phage hydrogels will also excel in the cultivation of organoids in the future.

In addition, 3D bioprinting, microfluidic, organoid fusion and organ-on-a-chip are also considered to be highly potential organoid culture techniques. Among these emerging methodologies, microfluidic technology facilitates downstream information detection, organ-on-a-chip enables scalable organoid generation, 3D bioprinting also demonstrates particular promise in advancing organoid technology in regenerative medicine through two distinctive contributions: (a) precise spatial reconstruction of tumor-stromal interfaces via layer-by-layer deposition of bioinks containing cancer cells, fibroblasts and endothelial cells [181]; (b) generation of heterogeneous tumor models through multi-nozzle printing that recapitulates intratumoral genetic diversity [182]. Three-dimensional bioprinting enables spatiotemporal regulation of hydrogel characteristics through dynamic control of mechanical properties and geometric constraints during culture evolution. In this model, engineered hydrogel confinement not only promotes cell polarity in hepatocarcinoma but also directs enteroid carcinoma morphogenesis, demonstrating critical capacity for enhancing tumor organoid maturation [183]. The integration of organoid culture techniques holds promise to address the limitations (e.g. low clinical relevance, high heterogeneity and immune rejection) of engineered organs in clinical applications and advance the progress of regenerative medicine [184]. In the field of regenerative medicine and organoid engineering, organoids derived from adult or pluripotent stem cells and cultivated via emerging technologies such as 3D bioprinting and microfluidics demonstrate clinical potential for repairing brain, skin, bone, liver and intestinal tissues [185, 186]. Future applications will further highlight the transformative role of organoids in advancing regenerative therapies. Moreover, organoids have now been developed or adopted for preclinical testing by biotechnology companies [187]. In parallel, a nonprofit organization collected all well characterized organoids generated from patient tissues and established a living biobank named hub (Hubrecht Organoid Technology), which has great implications for systematically conducting drug development and personalized medicine [178].

However, although the use of organoids also solves some biological and pharmacological problems, the road towards a broad-ranging translation of organoid technology into preclinical and clinical applications remains challenging. To overcome these challenges, various novel protocols are being developed, such as strategies to more accurately model organs and associated diseases by incorporating immune or mesenchymal cells and bioengineering strategies to obtain morphologically homogeneous organoids [188]. Given the rapid technological advances in the field, we believe that highly accurate and reproducible culture models will emerge that will overcome the current limitations that hinder the clinical transition of organoids and accelerate our comprehension of human development, disease and therapy.
